# Structural Diversity in the Dandelion (*Taraxacum officinale*) Polyphenol Oxidase Family Results in Different Responses to Model Substrates

**DOI:** 10.1371/journal.pone.0099759

**Published:** 2014-06-11

**Authors:** Mareike E. Dirks-Hofmeister, Ratna Singh, Christine M. Leufken, Jennifer K. Inlow, Bruno M. Moerschbacher

**Affiliations:** 1 Department of Plant Biology and Biotechnology, Westphalian Wilhelms-University of Münster, Münster, Germany; 2 Department of Chemistry and Physics, Indiana State University, Terre Haute, Indiana, United States of America; National Institute for Medical Research, Medical Research Council, London, United Kingdom

## Abstract

Polyphenol oxidases (PPOs) are ubiquitous type-3 copper enzymes that catalyze the oxygen-dependent conversion of *o*-diphenols to the corresponding quinones. In most plants, PPOs are present as multiple isoenzymes that probably serve distinct functions, although the precise relationship between sequence, structure and function has not been addressed in detail. We therefore compared the characteristics and activities of recombinant dandelion PPOs to gain insight into the structure–function relationships within the plant PPO family. Phylogenetic analysis resolved the 11 isoenzymes of dandelion into two evolutionary groups. More detailed *in silico* and *in vitro* analyses of four representative PPOs covering both phylogenetic groups were performed. Molecular modeling and docking predicted differences in enzyme-substrate interactions, providing a structure-based explanation for grouping. One amino acid side chain positioned at the entrance to the active site (position H_B2_+1) potentially acts as a “selector” for substrate binding. *In vitro* activity measurements with the recombinant, purified enzymes also revealed group-specific differences in kinetic parameters when the selected PPOs were presented with five model substrates. The combination of our enzyme kinetic measurements and the *in silico* docking studies therefore indicate that the physiological functions of individual PPOs might be defined by their specific interactions with different natural substrates.

## Introduction

**Figure 1:**
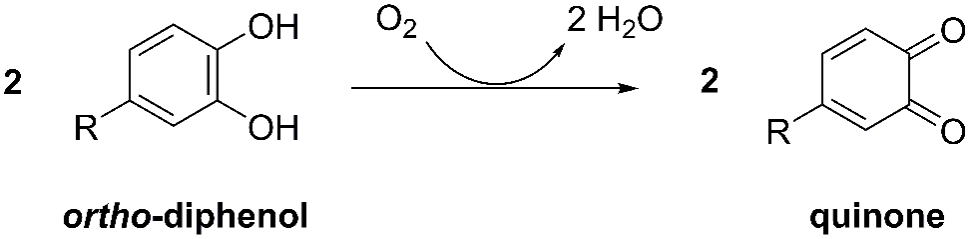
Enzymatic conversion of *ortho*-diphenols to the corresponding quinones by PPOs.

Polyphenol oxidases (PPOs, EC 1.10.3.1) are type-3 copper enzymes that catalyze the oxidation of diphenols to their corresponding quinones with molecular oxygen as a co-substrate (Figure 1). PPOs are found throughout the plant kingdom and their physiological functions are diverse, e.g. many are involved in responses to biotic or abiotic stress but this is not the case for every PPO in every plant species [1]–[6]. Plant PPOs are almost always present as multiple isoenzymes, differing in catalytic activity and expression profile, potentially corresponding to their distinct biological functions [7]–[11]. Little is known about the sequence–structure–function relationship among plant PPO isoenzymes due to the lack of a suitable expression system. Data from previous studies is therefore wholly derived from the sequencing and expression patterns of genes or the activity and characterization of partially-purified enzymes.

Comparative sequence analyses show all plant PPOs to have in common a three-domain structure [4], [12]–[13]. PPO sequences are prefixed by a transit peptide, locating the mature PPO-proteins inside the thylakoid lumen of chloroplasts [14]. Mature PPOs consist of the active-site-containing tyrosinase domain, a short linker region, and a C-terminal domain hypothized to cover the active site of the enzyme when in its latent state [13], [15]–[16]. *In vitro* activation of the latent enzyme can be accomplished by addition of SDS or proteolysis to remove the C-terminal domain [17]–[19]. Although some PPOs function as monomers [16], there are reports of multimeric forms that show enzymatic cooperativity [10], [14], [20], [21]–[22]. Two plant PPO structures have been solved by crystallography so far, and both structures comprise the tyrosinase domain only [23]–[24]. The natural substrates for most plant PPOs are unknown, although diphenolic flavonoids such as catechins have been proposed [25].

Here we investigated the structure–function relationship among plant PPOs by comparing purified recombinant dandelion (*Taraxacum officinale*) PPO isoenzymes using both experimental assays and a molecular modeling approach. Although dandelion is not a typical model organism, our experiments were motivated by the unique structural and functional diversity of the dandelion PPO family. We compared latex PPOs that are known to promote the coagulation and browning of dandelion milk sap [12] to isoenzymes PPO-2 which is required for resistance to *Pseudomonas syringae* pv. *tomato* [11] and PPO-6 which functions as a cooperative tetramer (previously was modeled in [20]). In addition we identified, sequenced and characterized a set of additional dandelion PPOs.

Phylogenetic analysis, enzymatic characterization, molecular modeling and *in silico* substrate docking showed that the dandelion PPO family resolves into two distinct groups with significant structural differences in the catalytic pocket, correlating with differing responses to model substrates. These data lead us to propose that differing specificities for their as-yet-undetermined natural substrates may make the physiological functions of specific isoenzymes distinct, thus providing a rationale of more detailed investigations of the structure–function relationships within the plant PPO family.

## Materials and Methods

### Identification and Sequencing of Novel PPO Genes

A PCR-based approach was developed to identify novel PPO genes using consensus-degenerate hybrid oligonucleotide primers (CODEHOP) [26]. Two primers – derived from the conserved CuA and CuB sites, respectively – were designed using an alignment of several plant PPO sequences: CuA_fw, 5′-GCA GGT GCA CAA CTC CTG GYT NTT YYT NCC-3′, and CuB_bw, 5′-GCG GAG TAG AAG TTG CCC ATR TYY TC-3′.

Touchdown PCR was carried out on genomic DNA and cDNA from different dandelion tissues. The stringency of the PCR conditions was reduced successively until no more new PPO sequences were found. The PCR products were sequenced and motifs were identified using the Pfam database [27]. Whenever a tyrosinase motif was found, the corresponding complete gene sequence was isolated using the Universal GenomeWalker kit (Clontech, Mountain View, CA, USA) and verified by proofreading PCR.

### Sequence and Expression Analysis

The full-length amino acid sequences of the PPO-isoenzymes 1–11 were analyzed as previously described [13]. RNA extraction and cDNA synthesis [11] was followed by the analysis of PPO gene expression using semi-quantitative reverse transcriptase (RT)-PCR with gene-specific primers to investigate (a) gene expression in different tissues; (b) gene expression in response to model infections (*Botrytis cinerea* and *Pseudomonas syringae* pv. *tomato*) [11] and spider mite infestation; and (c) gene expression in response to methyl jasmonate elicitation.

### Phylogenetic Analysis

The amino acid sequences of mature dandelion PPOs 1–11 (excluding the transit peptide) were aligned using MUSCLE v3.7 configured for highest accuracy (default settings) [28]. The phylogenetic tree was constructed using the maximum likelihood method implemented in Phylemon v2.0 [29]. The reliability of internal branches was assessed by bootstrapping (1000 replicates). The phylogenetic tree was visualized using MEGA v4.0.1 [30].

### Molecular Modeling

**Figure 2:**
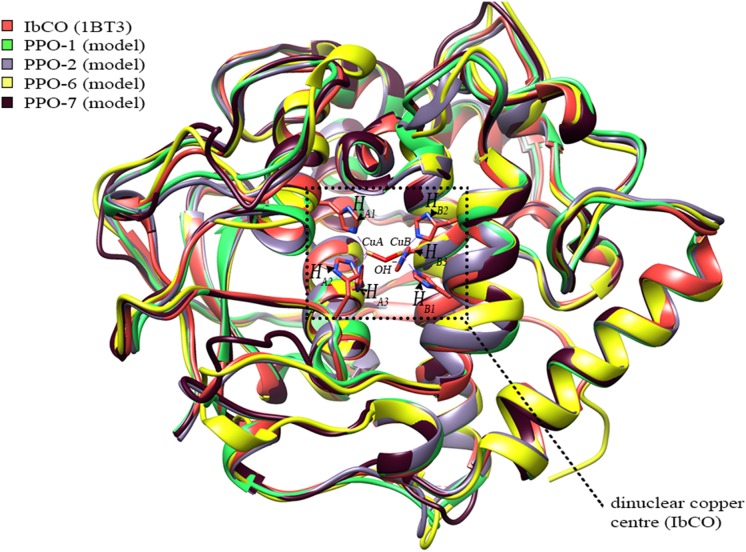
Superposition of the dandelion PPO models with the modeling template. 3D models of the tyrosinase domains of PPOs 1, 2, 6, and 7 were generated using the I-Tasser online structure prediction tool [31]. The crystal structure of IbCO (PDB 1BT3) [23] was used as the template for structure prediction. The coordinating histidine residues of IbCO (H_A1–A3_ and H_B1–B3_) were drawn and labeled accordingly, as well as the bound Cu-ions (CuA and CuB) and the hydroxyl ion (OH^−^) of the *met*-form.

3D models of the tyrosinase domains of PPOs 1, 2, 6, and 7 were generated using the I-Tasser online structure prediction tool [31]. The C-terminal domains were not modeled. The crystal structure of the sweet potato (*Ipomoea batatas*) catechol oxidase (IbCO, PDB 1BT3) [23] served as a template (Table 1). RMSD values were calculated for the generated models compared to the reference structure (PDB 1BT3) using the UCSF-chimera molecular modeling program (Table 1, Figure 2) [37]. Energy minimizations and stereochemical corrections of the constructed structural models were carried out using the KoBaMIN server [32]–[33] and geometric accuracy of the refined models were evaluated using Molprobity 4.02 [34]. This program performs numbers of validations resulting in a Molprobity score that provides an assessment of the final quality of the models (Table 1). As these scores were >70% for the generated models of PPOs 1, 2, 6, and 7, we deemed the quality good for docking studies.

**Table 1:** Evaluations of the molecular models generated for PPOs [23], [32], [34].

protein model	PPO-1	PPO-2	PPO-6	PPO-7
sequence identity [%] compared to the template	58	59	50	53
RMSD [Å] of the generated model^a^	0.492	0.544	0.601	0.538
starting amino acid for modeling	D95	D92	D90	A89
ending amino acid for modeling	Y424	Y421	Y430	Y433
KoBa potential energy [kcal/mol]	−6259	−6548	−5555	−7160
Clash score^b^	8.29	5.93	10.9	9.15
Outliers^c^ [%]	0.30	1.22	1.99	2.62
Favored^d^ [%]	95.43	95.73	93.45	94.75
Poor rotamers [%]	2.11	0.71	0.99	1.69
Bond length/bond angles^e^ [%]	0	0	0	0
Cβ^f^	0	0	0	0
Molprobibty scores [%]	75	92	77	75

a RMSD was calculated with the UCSF-chimera molecular modeling program [37] using all Cα atoms for calculation.

b Number of clashes per 1000 atoms.

c Percentage of residues with phi-psi angle combinations that lie in the disallowed region of the Ramachandran plot.

d Percentage of residues with phi-psi angle combinations that lie in the favored region of the Ramachandran plot.

e Percentage of backbone bond length/bond angles >4 standard deviations from the accepted values.

f Number of residues with Cβ deviations ±0.25 Å.

As PPOs are cofactor dependent proteins providing a dinuclear copper centre, the copper ions had to be introduced into the models. Therefore, the 3D xyz coordinates of the Cu-O-Cu position (*met* form) were retrieved after superimposition of the models to the inhibitor bound form of the template (PDB 1BUG, A), and introduced into the modeled structures of PPOs 1, 2, 6, and 7. We modeled the *met* form of the active site as being one state of substrate binding in the proposed catalytic cycle of PPOs [35]. The copper-coordinating histidine residues were then optimized to coordinate with the introduced Cu ions.

### Docking Studies

For docking studies, the 3D structures of the model substrates were obtained from the Pubchem database [36] and Chimera was used to assign charges to substituted carboxylate and amino groups to generate sybyl MOL2 format structure files [37]. Docking was carried out using AutoDock v4.2 [38]. Hydrogen atoms were added to substrate and protein structures, non-polar hydrogens were merged, and flexibility was assigned to small molecules by setting the number of torsions. The docking grid size was prepared using the *autogrid* utility. The grid center was adjusted such that the grid boxes included the entire cavity of the PPO active site, providing enough space for ligand translational and rotational walks. The best conformations were sought using the Lamarckian genetic algorithm (LGA).

During the docking process, a maximum of 100 conformations was considered for each compound. The population size was set to 150 and individuals were initialized randomly. The maximum number of generations was set to 27,000 with 2,500,000 evaluations. The maximum number of top individuals that automatically survived was set to 1, the mutation rate was set to 0.02 and the crossover rate was set to 0.80. After docking, the binding energy of the substrate and active site was evaluated using the *autoscorer* utility, which considers the hydrogen bond forces as well as electrostatic forces, van der Waals forces, solvation energy and entropy [38].

### Heterologous Expression and Purification of Recombinant Proteins

Genes of PPOs 1, 2, 6 and 7 (without the sequence encoding the transit peptide) were selected and supplemented with an N-terminal Strep2 tag (WSHPQFEK) including the enterokinase recognition site (DDDDK) as a spacer. The genes were then transferred to pET22b(+) (Novagen, Merck, Germany), introduced into *Escherichia coli* Rosetta2 (DE3) pLysSRARE2 cells (Novagen) and verified by sequencing.

Recombinant PPOs 1, 2, 6, and 7 were expressed in autoinduction medium [39] supplemented with 20 µM CuCl_2_ at 26°C. The cells were grown for 48 h until the OD_600 nm_ was ∼3, then they were pelleted and lysed, and the intracellular fractions were harvested by centrifugation. The proteins were purified by StrepTactin (IBA, Germany) affinity chromatography and quantified using the Bradford assay [40]. The purified enzymes were stored at 4°C in 50 mM maleate-Tris buffer (pH 6) supplemented with 1 M sucrose and threefold the PPO content of bovine serum albumin for downstream enzymatic analysis. For short-term storage prior to SDS-PAGE analysis, the enzymes were solved and stored in 50 mM maleate-Tris buffer (pH 6.0) without supplements and incubated at 4°C.

### SDS-PAGE Analysis

The purity of the recombinant enzymes was determined by SDS-PAGE using 12% acrylamide gels [41] and a non-reducing loading-dye (62.5 mM Tris-HCl pH 6.8, 2% SDS, 5% glycerol, 0.04% bromophenol blue). After electrophoresis, the proteins were stained with 0.1% Coomassie Brilliant Blue G-250 in 40% methanol, 5% acetic acid.

### PPO Activity

**Figure 3:**
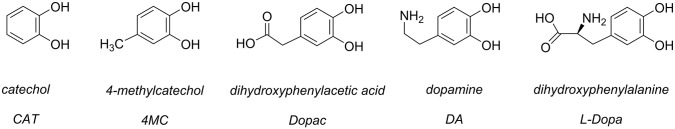
Structures of the diphenolic substrates used for kinetic analysis.

The following *o*-diphenolic compounds were used as model substrates for PPO (Figure 3): catechol (CAT, benzene-1,2-diol), 4-methylcatechol (4MC, 4-methylbenzene-1,2-diol), dihydroxyphenylacetic acid (Dopac, 2-(3,4-dihydroxyphenyl)acetic acid), dihydroxyphenylalanine (L-Dopa, (S)-2-amino-3-(3,4-dihydroxyphenyl)propanoic acid) and dopamine (DA, 4-(2-aminoethyl)benzene-1,2-diol).

The appearance of corresponding quinones generated by PPO activity was monitored at specific wavelengths as listed in Table 2. The molar extinction coefficients for these quinones were determined in 50 mM acetate-phosphate buffer at pH 5.0 as previously described [42]. In brief, various amounts of substrate were chemically oxidized by addition of a 10-fold concentration of NaIO_4_, and the quinone formation and stability were monitored spectrophotometrically at several time points. The extinction coefficient was then determined by linear regression of amount of product onto measured absorption at the appropriate wavelength.

**Table 2:** Molar extinction coefficients (e_x nm_) of PPO substrates determined at pH 5 [42].

substrate	wavelength [nm]	e_x nm_ [M^−1^ cm^−1^]
CAT	400	1197
4MC	405	1090
Dopac	410	1181
DA	400	1039
L-Dopa	400	1112

PPO activity was measured at 3-s intervals over a duration of 2 min in 50 mM acetate-phosphate buffer at 25°C. The buffer was adjusted for the optimal pH of each enzyme by the addition of suitable amounts of NaOH. PPO activity was determined in triplicate using 5 nM of purified enzyme for each individual measurement and was calculated from the maximum slope of the product-formation curves by fitting a linear regression of seven measured points using SoftMax Pro Software (Molecular Devices, USA).

### Enzyme Kinetics

PPO activities were plotted against the substrate concentrations and the Michaelis-Menten equation was fit to the data using the non-linear regression feature in SigmaPlot v11.0.

### Statistical Analysis of Group Parameters

Kinetic measurements were taken for three independent batches of each isoenzyme including several repeated measurements for all five substrates. The overall group 1 and group 2 means (n = 70 and n = 84) of the parameters were calculated and the standard errors for mean K_m_, k_cat_ and k_cat_/K_m_ values across all substrates for groups 1 and 2 were calculated using error propagation formulas. We then used the standard normal distribution to compute the p-value, i.e. a *z* test instead of a *t* test. Further information can be found in the Statistical Analysis Supplement (Text S1).

## Results

### Extension of the PPO Dandelion Gene Family

**Figure 4:**
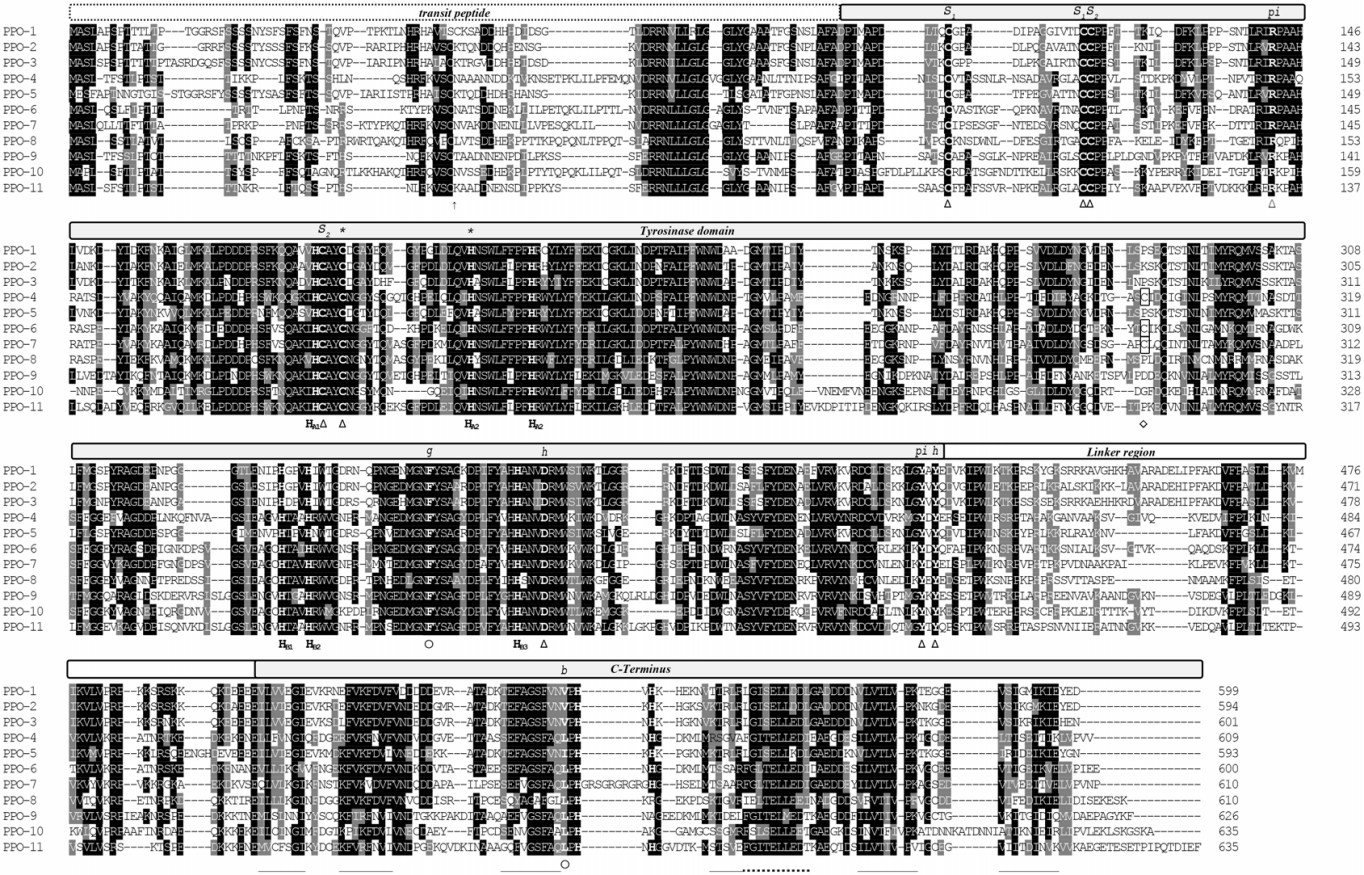
Sequence analysis of the dandelion PPO family. Amino acid sequences of all PPOs were aligned using MUSCLE v3.7 [28]. Identical and similar residues are shaded in black and gray, respectively. Domain structure was analyzed [13], [43]–[44] and findings were marked as follows. The predicted transit peptide is labelled and the predicted cleavage site of the stromal processing peptidase is marked with an arrow. The catalytic tyrosinase domain, linker region, C-terminal domain, and copper-binding histidines of the CuA (H_A1_–H_A3_) and CuB (H_B1_–H_B3_) sites are labelled. Structurally important residues are marked by triangles (Δ) and potentially regulative residues by circles (○). The predicted β-strands of the conserved β-sandwich C-terminal domain are marked by straight underlining ( _ ) and the conserved helix by dotted underlining (…). Cysteines of potential multimerization sites [20] are boxed and their position is marked by a diamond (◊). Cysteines potentially involved in two intramolecular disulfide bonds are labeled S_1_ and S_2_. [S, disulfide linkage; *, thioether bridge; h, hydrogen bond; pi, π-cation interaction; g, gate residue; b, blocking residue].

Six genes, namely PPOs 1 to 6, encoding dandelion PPOs have already been sequenced and described [11]–[12], [20]. However, the amount of browning in protein extracts from a variety of dandelion tissues suggested that additional PPO genes may be present. We therefore used a PCR approach with degenerate primers and different stringency conditions to amplify PPO-like sequences from dandelion genomic DNA and cDNA from various tissues. This led to the identification of five additional PPO genes, namely PPOs 7 to 11, which were then fully sequenced and characterized in terms of protein targeting, molecular weight and isoelectric point (Table 3). Sequence analysis was then used to determine the domain structure and to locate structurally and catalytically important amino acid residues, as previously described [13] (Figure 4).

**Table 3:** Overview of the PPO family in dandelion.

notation	size		mature protein			identity^c^	observed expression^d^	miscellaneous
	gene	ORF	cellular localization^a^	M_W_ b	pI^b^			
PPO-1	1800 bp	599 aa	C (cTP 0.931, RC 1)	57.0	5.5	100	*constitutive in latex and root*	major latex protein [12]
PPO-2	1785 bp	594 aa	C (cTP 0.924, RC 3)	56.8	6.1	79.8	*infection-induced in leaf; constitutive in root*	specific function in resistance [11]
PPO-3	1806 bp	601 aa	C (cTP 0.928, RC 1)	57.0	7.4	81.8	*occasionally in latex*	-
PPO-4	1830 bp	609 aa	C (cTP 0.799, RC 2)	57.8	5.5	46.4	*occasionally in leaf; constitutive in stalk and flower*	potential tetramerization
PPO-5	1787 bp	593 aa	C (cTP 0.859, RC 2)	56.7	5.8	70.3	*-*	-
PPO-6	1803 bp	600 aa	C (cTP 0.814, RC 2)	57.8	6.0	45.2	*methyl jasmonate induced in leaf; constitutive in stalk and flower*	tetrameric PPO [20]
PPO-7	1833 bp	610 aa	C (cTP 0.869, RC 2)	58.3	5.8	47.2	*constitutive in latex, leaf and root; repressed by infections in leaf*	tetramerization observed
PPO-8	1833 bp	610 aa	C (cTP 0.914, RC 1)	59.0	5.7	44.1	*-*	-
PPO-9	1881 bp	626 aa	C (cTP 0.835, RC 2)	61.5	5.8	43.4	*occasionally in leaf*	-
PPO-10	1908 bp	635 aa	C (cTP 0.825, RC 2)	61.8	6.1	41.5	*constitutive in latex, leaf, root, stalk and flower*	-
PPO-11	1908 bp	635 aa	C (cTP 0.612, RC 3)	62.9	5.9	43.2	*-*	-

a Predicted by the TargetP online tool [43]–[44]. C: predicted location is the Chloroplast; cTP: chloroplast transit peptide score, from 0 to 1 where 1 indicates the highest likelihood; RC: Reliability class, from 1 to 5, where 1 indicates the strongest prediction. Due to the twin-arginine motif (Figure 3) localization is predicted to be in the thylakoid lumen.

b Calculated average molecular weight (M_W_) in kDa and isoelectric point (pI) of the mature protein (without the predicted transit peptide), computed by the Compute pI/Mw tool [45]–[46].

c Values given in % sequence identity of full length protein sequences relative to PPO-1 (sequence identity matrix created by ClustalW2.1).

d Expression was repeatedly tested by semi-quantitative RT-PCR; PPOs which were expressed in a given tissue in at least one experiment were assigned as “occasionally” expressed.

### Dandelion PPOs Comprise Two Distinct Groups

**Figure 5:**
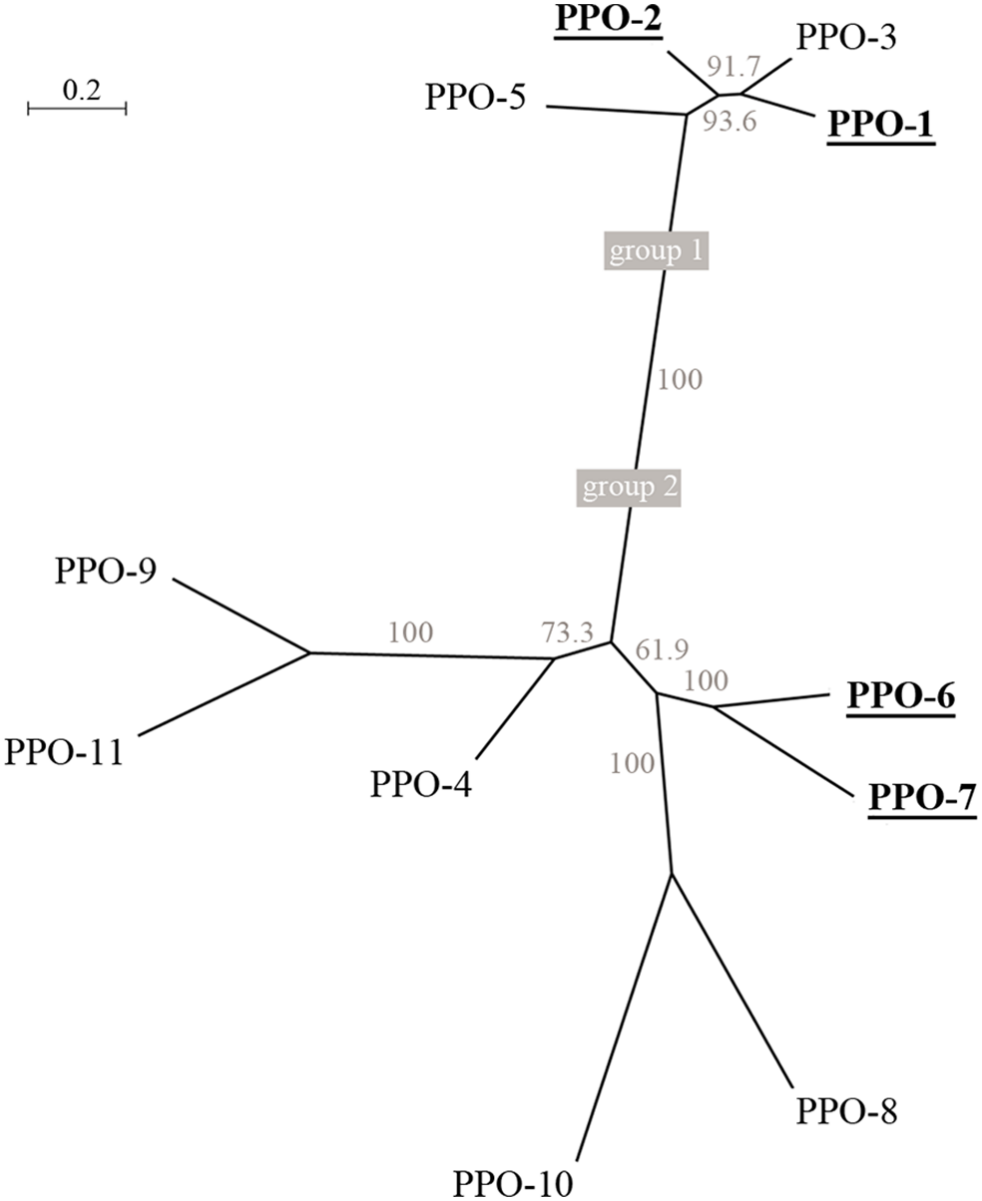
Phylogenetic tree of the dandelion PPO family. PPO amino acid sequences were aligned using MUSCLE v3.7 [28]. The phylogenetic tree was constructed using the maximum likelihood method implemented in Phylemon v2.0 [29]. The reliability of internal branches was assessed using the bootstrap method (1000 replicates). PPOs selected for further characterization are underlined.

The PPO sequence data indicated an unexpectedly high level of diversity within the dandelion PPO family. Although the domain structure, chloroplast localization signal and certain structurally-important residues (e.g., the so-called blocking residue, four disulfide-forming cysteines, the thioether-forming cysteine-histidine pair, and the Cu-coordinating histidines [13]) are conserved in all PPOs (Figure 4); differences in the sequences immediately flanking the first and second CuB-coordinating histidine residues (H_B1_ and H_B2_) were found to correlate with a separation of the PPOs into two distinct phylogenetic groups (Figure 5). PPOs 1–3 and 5 were assigned to group 1, and all the other PPOs (4 and 6–11) were assigned as group 2. We selected four PPOs (PPOs 1, 2, 6, 7), two representing each phylogenetic group, for more detailed *in silico* and *in vitro* analysis.

### *In silico* Analysis of the Catalytic Pocket

**Figure 6:**
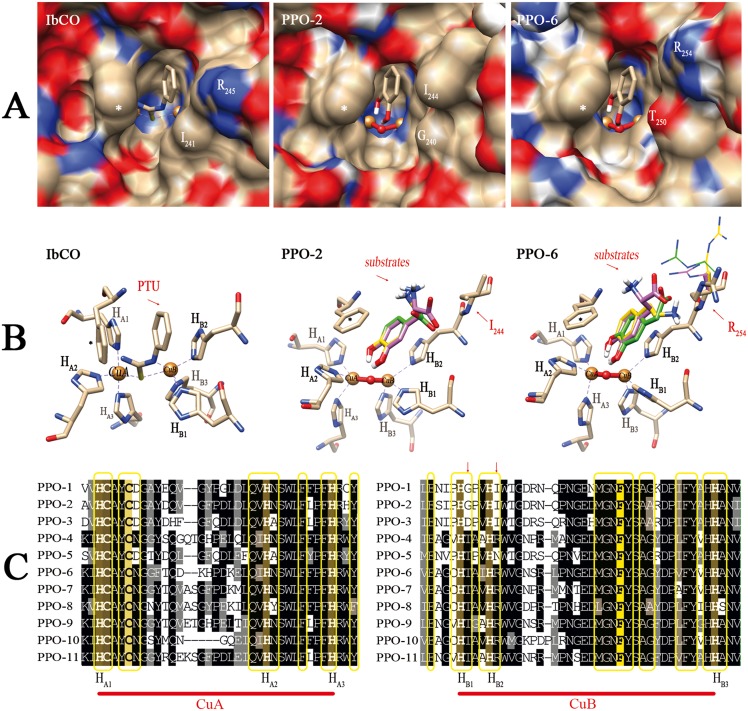
Molecular modeling and docking studies of dandelion PPOs. PPO-2 and PPO-6 are shown here as representatives of each phylogenetic group. Surfaces and stick models are colored according to atom type (blue, nitrogen; red, oxygen; gray, carbon; white, hydrogen) and the following special designations: * = gate residue (phenylalanine); gold spheres = copper atoms; red sphere = bound oxygen in met-form. (A) Surface contour images of the catalytic pocket, showing the crystallographic structure of IbCO (PDB 1BUG,A) and homology models of PPO-2 (group 1) and PPO-6 (group 2). The substrate analog phenylthiourea (PTU, *stick representation*) occupies the substrate binding site in the hydrophobic cavity. For comparison, the binding of CAT (*stick representation*) as the simplest substrate is shown in the active site for the modeled dandelion PPOs. Residues H_B1_+1 and H_B2_+1 are labeled at the entrance of the catalytic pocket. (B) Predicted interactions of the substrate in the active site resulting from docking analysis. The position and interaction of IbCO with PTU is shown as a reference. PPO-2 and PPO-6 are shown binding to Dopac (green), DA (yellow) and L-Dopa (purple); only polar hydrogen atoms are drawn for the substrates. For PPO-6, different R254 side chain rotamers resulted from binding of the different substrates. The rotamers are drawn as thin stick and are colored green, yellow or purple according to the corresponding substrate. (C) Sequence alignment of the copper-binding sites (CuA and CuB) of all eleven dandelion PPOs. Identical and similar residues are shaded in black and gray, respectively. The Cu-binding histidine residues (H_A1–A3_, H_B1–B3_) are labeled, and arrows indicate the H_B1_+1 and H_B2_+1 positions. The gate residue is shaded in yellow. Residues located within 8Å of the copper centers in 3D space are boxed in yellow.

Three-dimensional structures for PPOs 1, 2, 6, and 7 were modeled using the crystal structure of sweet potato (*Ipomea batatas*) catechol oxidase (IbCO, PDB 1BT3) as a template. The backbone conformations of the resulting models were very similar to that of the template (Figure 2, Table 1). The models were deemed to have sufficient quality for further studies, as analysis of the models showed that >93% of the residues were located in the favored region of a Ramachandran plot, while also having favorable rotamers, backbone bonds lengths and angles, and no significant Cβ deviations (Table 1). All models showed the four-helix bundle typical of homologous PPOs, tyrosinases, and catechol oxidases (Figure 2) as well as the dinuclear copper centre consisting of the six conserved histidines known to coordinate the two copper atoms (Figure 6). The structural alignment showed some loop regions to slightly differ between the models (Figure 2). Most of the amino acid residues in close spatial proximity to the active site are highly conserved (Figure 4, Figure 6), so it is expected that binding of the substrate’s phenolic ring is similar for the four dandelion PPOs. However, some significant differences at positions H_B1_+1 and H_B2_+1 were mapped to the opening of the catalytic pocket (Figure 6 A), and these differences correlate with the phylogenetic grouping of the dandelion PPO family (Figure 6 C). Accordingly, we predict dandelion group 1 (PPOs 1 and 2) to be characterized by an open and unrestricted entrance to the catalytic pocket with no charged side chains in close proximity, and in contrast group 2 (PPOs 6 and 7) to have a more restricted entrance due to the presence of a bulky, charged arginine at position H_B2_+1 (R254 for PPO-6, R258 for PPO-7, based on numbering that starts with the first amino acid of the mature PPO excluding the transit peptide).

In order to predict the influence of the H_B2_+1 position on the binding of different substrates, docking studies were carried out. Five different diphenolic substrates were used, these differing in the length and character of the substituent on the phenolic ring (Figure 3). In the docking simulations, 100 conformations were generated for each substrate and the best pose was selected based on minimum energy conformation (Table 4). The positioning of the docked substrate was then compared to the binding position of an inhibitor that is present in the crystal structure of IbCO (PDB 1BUG,A) as shown in Figure 6. Due to the restricted entrance to the active site for group 2 PPOs, two sets of docking simulations were carried out for PPOs 6 and 7. In one set, docking simulations were performed without giving any flexibility to the side chain of the arginine at position H_B2_+1, so that its position was held in the same rotamer as that of the corresponding arginine in the crystallographic structure of IbCO. In a second set of docking studies, side chain flexibility was allowed for this arginine (Figure 6B). The comparison of these two sets of experiments indicates that the flexibility of the arginine side chain strongly influences the *in silico* substrate-binding behavior of the group 2 PPOs by reducing the predicted binding energies for Dopac and L-Dopa (Table 4).

**Table 4:** Docking studies.

enzyme			*ΔG_b_ of the substrate [kcal/mol]* a				
			CAT	4MC	Dopac	DA	L-Dopa
*group 1:*	PPO-1		−3.92	−4.37	−3.79	−5.27	−4.57
	PPO-2		−4.06	−4.41	−3.85	−5.33	−4.70
*group 2:*	PPO-6	b	−4.08	−4.53	−4.49	−4.87	−4.87
		c	−*4.15*	−*4.54*	−*7.41*	−*4.63*	−*7.70*
	PPO-7	b	−4.32	−4.75	−4.54	−5.55	−5.06
		c	−*4.29*	−*4.72*	−*7.32*	−*5.23*	−*8.00*

a Docking studies were performed with AutoDock (version 4.2) [38] to predict the binding energy (ΔG_b_) of the substrate in the active site.

b The side chain of the arginine at position H_B2_+1 was kept fixed in the same position/rotamer as that observed for the corresponding arginine in the crystallographic structure 1BUG,A of IbCO (i.e., no flexibility was assigned to this residue during docking).

c For a second set of docking studies side chain flexibility for the arginine at position H_B2_+1 was allowed. The isoleucine at the corresponding position in group 1 PPOs was not given flexibility.

### Heterologous Expression and Purification of Recombinant PPOs

Next, we studied the *in vitro* characteristics of the four selected dandelion PPO isoenzymes. To this end, we produced recombinant PPOs 1 and 2 from group 1 and PPOs 6 and 7 from group 2 in *E. coli*, and characterized the purified enzymes to determine the optimal conditions for activity.

**Figure 7:**
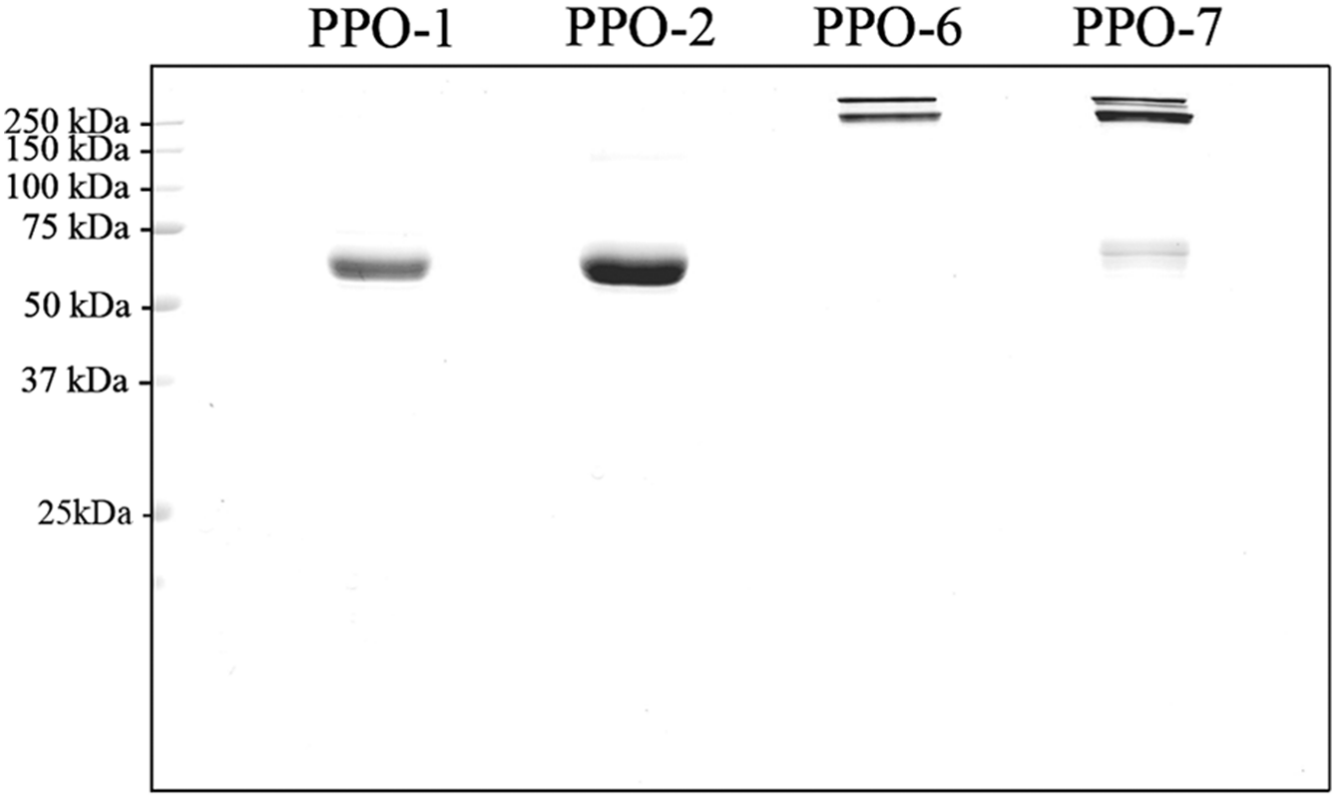
Purified recombinant PPOs. The gene sequences for dandelion PPOs 1, 2, 6 and 7 (excluding the transit peptide) were each supplemented with an N-terminal Strep2 tag using the enterokinase recognition site as a spacer. Proteins were purified by StrepTactin affinity chromatography and 3-µg samples were analyzed by SDS-PAGE using a non-reducing loading dye, followed by staining with Coomassie Brilliant Blue.

As anticipated based on the locations of cysteine residues (Figure 4), PPO-6 and PPO-7 showed a high molecular weight band on non-reducing SDS-PAGE gels (Figure 7) suggesting that both enzymes adopt a tetrameric quaternary structure stabilized by disulfide bonds, as previously demonstrated for PPO-6 [20]. Notably, both PPO-6 and PPO-7 show a double band on non-reducing SDS-PAGE which disappears under fully reducing, denaturating conditions (compare [20]). The appearance of a band at monomer molecular weight for PPO-7 under non-reducing conditions (Figure 7) suggests that the PPO-7 tetramer is less stable in SDS than the PPO-6 tetramer was shown to be [20].

### Optimal Conditions for PPO Activity

**Figure 8:**
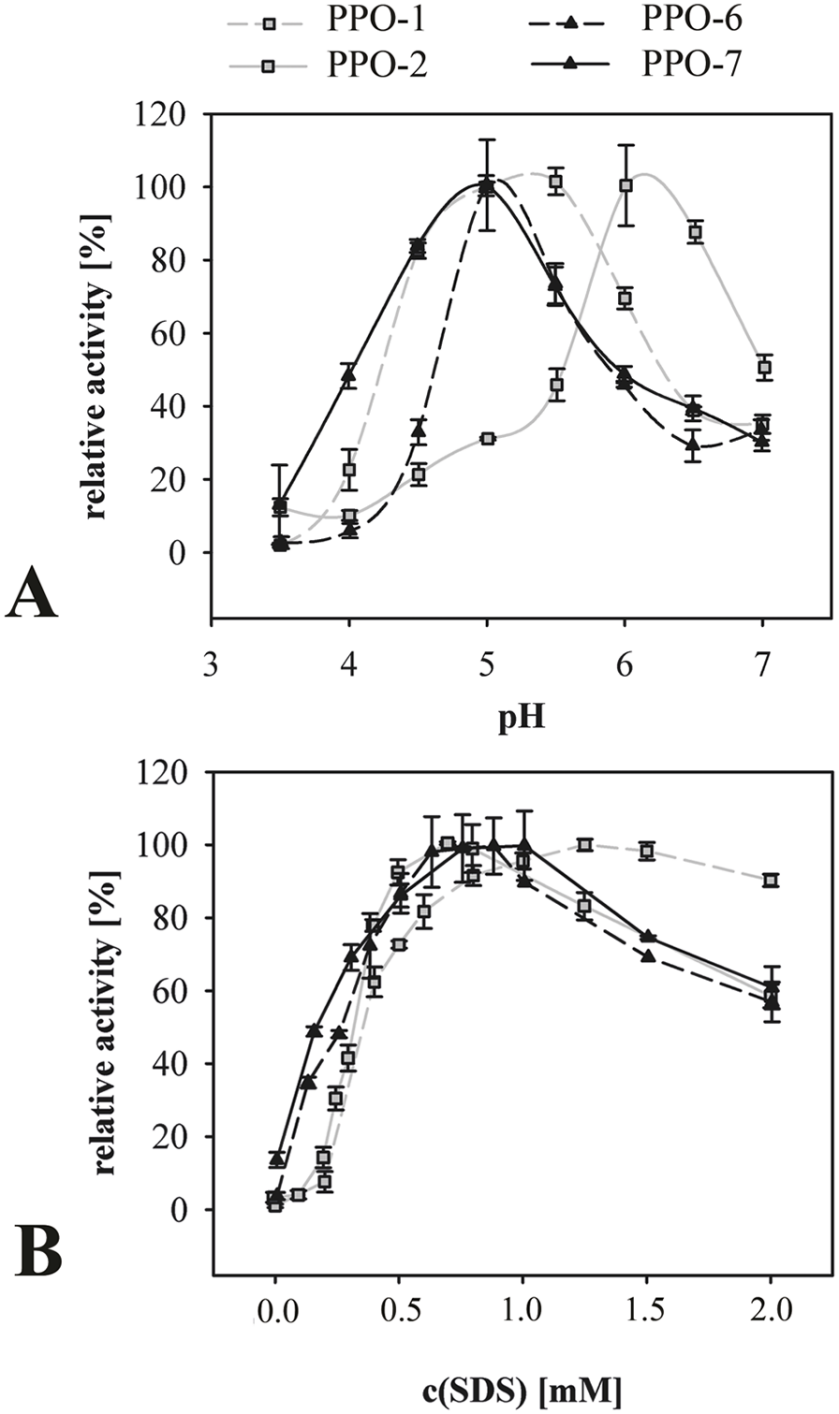
Optimal conditions for PPO activity. The activity of purified recombinant PPOs was monitored by spectrophotometry at 405-methylcatechol. (A) The influence of pH on PPO activity was determined using 100 mM acetate-phosphate buffer with the pH adjusted by adding NaOH. Activities were measured in the presence of 0.75 mM SDS (PPOs 2, 6 and 7) or 1.0 mM SDS (PPO-1). (B) Influence of SDS concentration on PPO activity. All measurements were taken at the optimal pH for each PPO (pH 5.0 for PPO 6 and 7, pH 5.5 for PPO-1, and pH 6.0 for PPO-2). All values are means ± standard deviation from one representative experiment measured in triplicate.

Plant PPOs can be activated by the addition of SDS [17]–[19] so we determined the optimal pH for each enzyme in the presence of a standard amount of SDS (Figure 8A) and then the optimal SDS concentration under these defined optimal pH conditions (Figure 8B). The four recombinant dandelion PPOs were characterized by differing optimal pH, which was thus adopted in later kinetic analysis experiments. PPO enzymes are often characterized by a sigmoidal SDS-dependent activity curve with concentrations above an optimum inhibiting the enzyme [17]–[19]. Each of the four recombinant dandelion PPOs showed the anticipated sigmoidal response (Figure 8B) and we therefore adopted the optimal SDS concentration for each enzyme in later kinetic analysis experiments.

### Enzyme Kinetics

We tested the recombinant PPOs 1, 2, 6, and 7 against several potential substrates (e.g. caffeic acid, quercetin, resveratrol, chlorogenic acid and protocatechuic acid) but none of them were processed by the recombinant PPOs (*data not shown*). Therefore, we used the five diphenolic model substrates (Figure 3) which had also been used in the docking studies and to which all four recombinant PPOs showed moderate activity. The values of the catalytic rate (k_cat_), Michaelis constant (K_m_) and catalytic efficiency (k_cat_/K_m_) were determined using repeated measurements for three independent batches of enzyme (Table 5), with each measurement made at the optimal pH and SDS concentration for a given PPO. The kinetic parameters were determined by fitting the Michaelis-Menton equation to the data by non-linear regression. A certain degree of cooperativity has been reported for PPO-6 [20] and was also discovered here for PPO-7. Nevertheless, the Michealis-Menten equation was used to fit kinetic data for all PPOs because the Hill coefficient for PPOs 6 and 7 was close to 1 (minimal cooperativity), resulting in only minor differences in kinetic parameters when using the Michaelis-Menten equation compared to the Hill equation. We preferred the Michaelis-Menten over the Hill equation as the former produces more robust kinetic parameters than the latter. Repeated measurements of independent batches of PPOs 1, 2, 6 and 7 produced similar results.

**Table 5:** Kinetic parameters for recombinant PPOs.

kinetic parameter^a^	PPO	substrate				
		CAT	4MC	Dopac	DA	L-Dopa
K_m_[mM]	PPO-1	1.19±0.51	1.06±0.18	4.95±1.67	0.78±0.14	8.07±2.14
	PPO-2	1.28±0.38	1.13±0.16	3.40±0.62	0.94±0.31	6.09±1.35
	PPO-6	5.57±0.47	4.71±0.39	2.33±0.60	5.62±1.14	13.45±4.70
	PPO-7	4.51±0.54	5.85±0.69	22.83±4.84	4.49±2.26	8.08±3.99
k_cat_[s^−1^]	PPO-1	31.6±2.0	165.4±16.4	81.4±14.7	78.9±6.5	67.7±11.7
	PPO-2	26.7±5.1	124.1±26.3	45.5±11.8	59.5±13.7	54.9±15.8
	PPO-6	304.1±43.0	514.0±72.0	50.7±10.0	61.7±10.6	64.3±19.8
	PPO-7	117.3±33.2	314.5±65.6	183.3±40.4	22.6±2.5	32.2±7.1
k_cat_/K_m_[s^−1^ M^−1^]	PPO-1	2.7±1.5*10^4^	15.6±2.3*10^4^	1.6±0.3*10^4^	10.1±2.0*10^4^	0.8±0.1*10^4^
	PPO-2	2.1±0.7*10^4^	11.0±2.9*10^4^	1.3±0.5*10^4^	6.3±2.8*10^4^	0.9±0.4*10^4^
	PPO-6	5.5±0.5*10^4^	10.9±1.3*10^4^	2.2±0.2*10^4^	1.1±0.1*10^4^	0.5±0.04*10^4^
	PPO-7	2.6±0.6*10^4^	5.4±1.3*10^4^	0.8±0.2*10^4^	0.5±0.4*10^4^	0.4±0.2*10^4^

a The values for kinetic parameters are presented as means ± SEM from the following number of measurements for each substrate: PPO-1 (n = 5), PPO-2 (n = 9), PPO-6 (n = 9), PPO-7 (n = 9).

**Figure 9:**
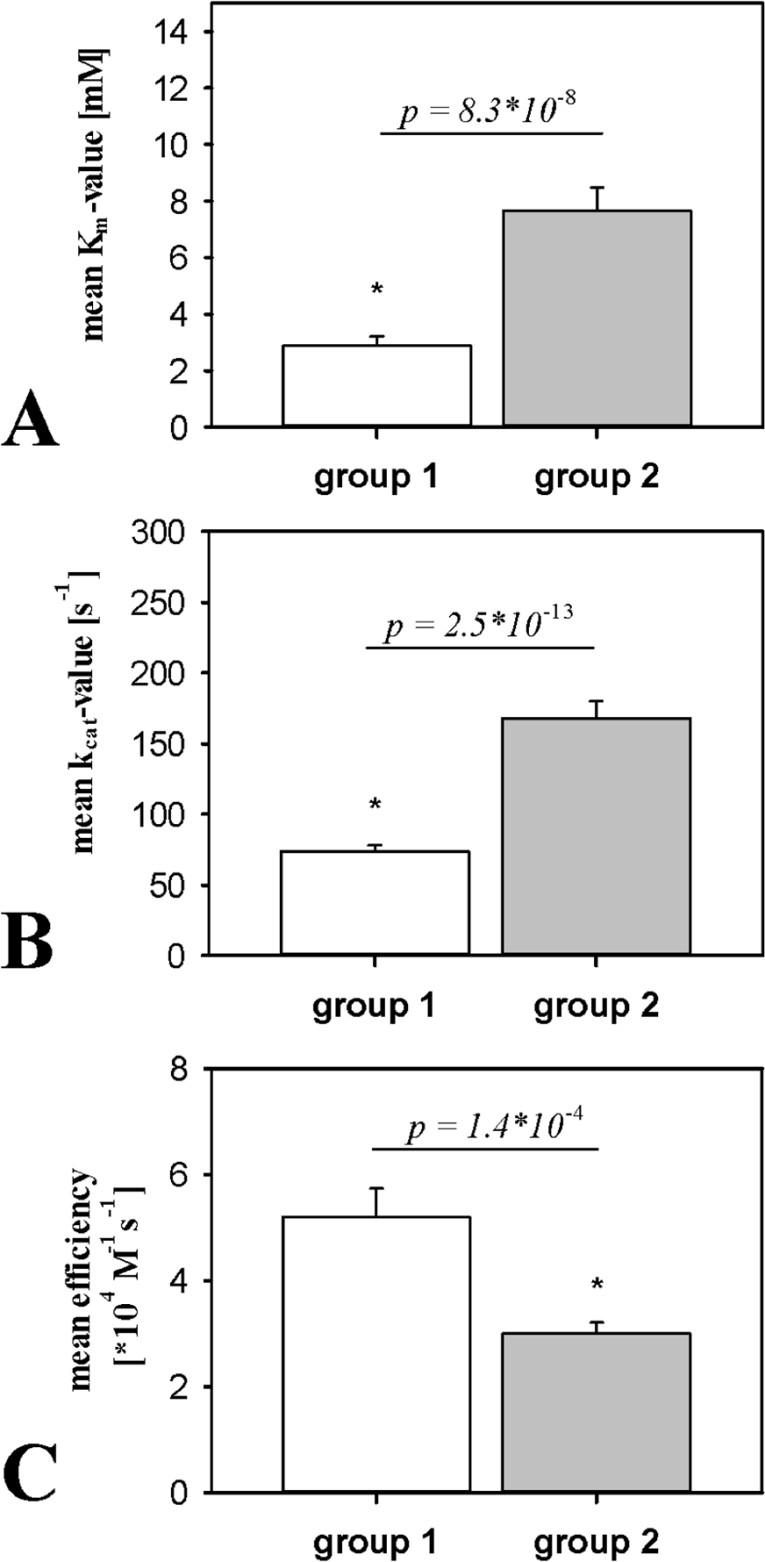
Comparison of kinetic parameters for the dandelion PPO groups. (A) Michaelis constants (K_m_ values). (B) Turnover rates (k_cat_ values). (C) Catalytic efficiencies (k_cat_/K_m_ ratios). The group values for kinetic parameters were calculated from original data (n = 70 for group 1, n = 84 for group 2) as means ± SEM using error propagation. The two groups were compared in terms of K_m_, k_cat_ and efficiency, and are indicated on the bar graphs as p-values determined in z-tests.

The kinetic parameters of the isoenzymes revealed some striking differences between the phylogenetic groups, as shown by the mean values for each group ± SEM given in Figure 9. The average K_m_ value of group 1 (PPOs 1 and 2, 2.89±0.32 mM) was lower than that of group 2 (PPOs 6 and 7, 7.66±0.83 mM) as shown in Figure 9A. This small but statistically-significant difference may reflect the higher substrate binding affinity of group 1. The average k_cat_ value of group 1 (73.8±4.4 s^−1^) was also lower than that of group 2 (168.0±12.1 s^−1^) as shown in Figure 9B. This statistically-significant difference indicates a higher catalytic turnover rate for model substrates by group 2 enzymes.

The K_m_/k_cat_ ratios for each isoenzyme/substrate combination indicated that group 1 PPOs (k_cat_/K_m_ = 5.2×10^4^±0.5×10^4^ M^−1^s^−1^) are more catalytically efficient than group 2 PPOs (k_cat_/K_m_ = 3.0×10^4^±0.2×10^4^ M^−1^s^−1^) as shown in Figure 9C. The lower K_m_ (potentially higher substrate binding affinity) of group 1 PPOs therefore appears to outweigh the higher turnover rate of group 2 PPOs. The difference in catalytic efficiency, as well as its components K_m_ and k_cat_, are thereby correlated with the phylogenetic grouping of the PPOs.

## Discussion

### Dandelion has an Unusually Large and Diverse PPO Family

The dandelion PPO family was expanded in our investigation to now include eleven genes. Because the dandelion genome has not yet been fully sequenced, the presence of even more PPO genes cannot be ruled out. However, eleven genes already is a rather large family compared to other, fully-sequenced plants [47]. All the novel dandelion PPOs contain the previously-described structurally and catalytically important amino acids (e.g. the copper coordinating histidines or the cysteins involved in disulfide bounds [13]), implying that these are likely to be functional genes rather than pseudogenes. This was supported by expression analysis confirming gene expression of eight of the PPO genes, while the circumstances under which the remaining three are expressed remain to be established (Table 3). The strictly controlled and fine-tuned spatiotemporal regulation of the dandelion PPO gene family argues strongly in favor of distinct functional roles for the different isoenzymes.

The dandelion PPO family resolves into two distinct phylogenetic groups, suggesting group-specific differences in function. Indeed, based on the *in silico* analysis of PPO evolution, it was recently suggested that the complex evolutionary history of plant PPOs reflects diverse biological roles [47]. The phylogenetic grouping of dandelion PPOs correlates with significant sequence divergence in the region surrounding the CuB site. Based on the known crystal structure of IbCO bound to the substrate analogue inhibitor phenylthiourea (PDB 1BUG,A), the PPO substrate is thought to bind at the CuB site [23], [48]–[49]. The sequence differences among dandelion PPOs are therefore likely to influence the accessibility and/or binding of substrates to the catalytic pocket of the different isoenzymes. Similarly, two distinct classes of PPOs correlating with sequence differences in the CuB region were found in eggplant, suggesting a similar division based on discrete structural and functional roles [50].

### The Phylogenetic Groups have Different Catalytic Pocket Architectures and Kinetic Parameters

Based on the crystal structure of IbCO bound to a substrate analog (PDB 1BUG,A), the catalytic pocket was described as a “hydrophobic cavity” that interacts with the catechol ring of the substrate [23]. These hydrophobic interactions enable substrate binding – specifically, the substrate is stacked in a sandwich structure between the imidazole ring of H_B2_ and the phenyl ring of the so-called gate residue (F261 for IbCO) [23]. Our docking experiments showed that the binding position of the catechol ring did not differ between the five substrates studied herein, nor between the four PPO isoforms (Figure 6B).

Substrate docking studies using IbCO predicted that the arginine residue at position H_B2_+1 (R245), located at the entrance to the catalytic pocket, would stabilize the enzyme-substrate complex with 4MC due to hydrophobic interactions [51]. The PPO models (Figure 6) revealed that group 2 PPOs are similar to IbCO, in that a bulky arginine residue is present at position H_B2_+1, i.e. R254 for PPO-6 and R258 for PPO-7. In contrast, a smaller and more hydrophobic isoleucine residue occupies the same position in group 1 PPOs (I244 in PPOs 1 and 2). The presence of larger residues (e.g. a threonine at H_B1_+1 and arginine at H_B2_+1) in group 2 PPOs reduces the size of the opening to the hydrophobic cavity and active site. We propose that these differences in the cavity explain most of the observed differences in K_m_ and k_cat_ values between the two phylogenetic groups.

These modeled structural differences and measured kinetic differences may translate into real differences in substrate affinities and specificities *in vivo*. The results of the docking studies lend support to this hypothesis, because the predicted energy of substrate binding was affected strongly by interactions between the substituent on the catechol ring and the arginine side chain at position H_B2_+1. Group 2 PPOs showed distinct binding energies for the negatively-charged substrates Dopac and L-Dopa, depending on the flexibility assigned to the arginine side chain (Table 4). We therefore conclude that substrate binding is at least partly influenced by the presence and mobility of this arginine side chain in group 2 PPOs, promoting either attractive or repulsive interactions with the charged functional group on the substrate. Furthermore, we propose that the H_B2_+1 residue may play a key role in this process, acting as a selector to determine substrate specificity for different PPOs *in vivo*. This hypothesis is currently being addressed by testing the activities of different mutant variants of each PPO.

Our data indicate that the closest residues to H_B1_ and H_B2_ (including H_B2_+1) are likely to play a fundamental role in the substrate specificity of plant PPOs. Indeed, a recent comparative study showed that these sequences varied significantly among PPOs in different plant species and PPO isoenzymes within species [47]. The crystal structure of wine grape PPO (PDB 2P3X) was recently used as a template for the homology modeling of aureusidin synthase, a PPO homolog with strict substrate specificity that helps to control flower coloration, providing further evidence that the residues neighboring H_B1_ and H_B2_ contribute to substrate specificity [52]. This portion of the CuB site may be a hotspot for the evolutionary adaptation of plant PPOs based on their corresponding phenolic substrates.

### Does Substrate Specificity Correlate with Physiological Function?

Our combined data based on PPO sequences, expression patterns, enzyme kinetics, *in silico* substrate binding and catalytic pocket architectures, suggest that different dandelion PPOs have distinct functions. The expression profile is clearly important to ensure enzyme availability, but the structural differences between isoenzymes may hold the key for defining distinct physiological functions.

With the exception of the non-catalytic hemocyanins, specific physiological functions have been assigned to only a few type-3 copper proteins, including the synthesis of melanin pigments in mammals and of secondary metabolites such as aurones and betalains in plants [53]–[55]. Specific natural substrates have been identified in these cases, but natural substrates are as yet unknown for most PPOs including the dandelion enzymes discussed herein, although flavonoids such as catechins have been proposed [25]. The unstable products of PPO activity, highly reactive quinones, are implicated in the potential resistance-related effects of PPOs [1], [11], [49], [56]–[58]. Specificity for natural substrates determined by structural differences among isoenzymes is likely to be necessary in order to fine-tune the physiological functions of dandelion PPOs. Regardless of whether or not the function is related to defense (including latex coagulation) or secondary metabolism, the measured kinetic differences and predicted structural differences between the two phylogenetic groups could reflect different strategies in the evolution of PPO activity. The identification of natural substrates and their localization, reactivity and character is therefore an essential step in order to determine the precise physiological functions of dandelion PPOs.

## Supporting Information

Text S1 **Statistical Analysis Supplement.**
(PDF)
