# Supplementary material for: Structural Diversity in the Dandelion (Taraxacum officinale) Polyphenol Oxidase Family Results in Different Responses to Model Substrates
Source: PLoS One. 2014 Jun 11;9(6):e99759. doi: 10.1371/journal.pone.0099759 (PMC4053514; doi:10.1371/journal.pone.0099759)
Supplement: Text S1 — Statistical Analysis Supplement. (PDF) [file pone.0099759.s001.pdf]

Statistical Analyses Supplement

Calculation of standard errors using error propagation. Due to the design of the experiment it was necessary to use error propagation formulas to compute the standard errors of various averages of the means presented in Table 3. The standard error of an estimate is the standard deviation of the values of that estimate resulting from repeatedly replicating the associated experiment. Therefore the method used to calculate the standard error must be consistent with how the data was collected. In this study the five substrates analyzed (CAT, 4MC, etc.) were chosen to represent five categories or classes within the universe of all possible substrates. Similarly, the four PPO's analyzed were chosen to represent two groups or classes of PPO's. This being the case, the ten group 1 substrate-by-PPO means, e.g., CAT-PPO1 (1.19), CAT-PPO2 (1.28), etc., cannot be considered a simple random sample from among all possible substrate-by-PPO means. In other words, each time we replicate the experiment we would not get a different random set of ten substrate-by-PPO means. Instead we would re-estimate the same ten substrate-by-PPO means. Therefore the formula for calculating the standard error of the mean of a simple random sample of values is not applicable. (Using that formula yields SEM = 0.828 for the group 1 mean K_m.)

Instead of using the simple random sample formula, we calculate the standard error using error propagation. Let $\bar{K}_{m,1}$ denote the average of the ten group 1 substrate-by-PPO group means, $\bar{K}_{m,1,i}$, $i = 1, \ldots, 10$. Then

$$\begin{aligned} \bar{K}_{m,1} &= \frac{1}{10}(\bar{K}_{m,1,1} + \bar{K}_{m,1,2} + \cdots + \bar{K}_{m,1,10}) \\ &= \frac{1}{10}\bar{K}_{m,1,1} + \frac{1}{10}\bar{K}_{m,1,2} + \cdots + \frac{1}{10}\bar{K}_{m,1,10}. \end{aligned}$$

Let SE_1, SE_2, $\ldots$, SE_{10} denote the standard errors of the ten $\bar{K}_{m,1,i}$, respectively. Then, by a basic error propagation result for independent measurements, we have[1,2]

$$\begin{aligned} SE_{\bar{K}_{m,1}} &= \left[\left(\frac{1}{10}\right)^2 SE_1^2 + \left(\frac{1}{10}\right)^2 SE_2^2 + \cdots + \left(\frac{1}{10}\right)^2 SE_{10}^2\right]^{0.5} \\ &= \left[\left(\frac{1}{10}\right)^2 0.51^2 + \left(\frac{1}{10}\right)^2 0.18^2 + \cdots + \left(\frac{1}{10}\right)^2 1.35^2\right]^{0.5} \\ &= 0.318. \end{aligned}$$

Calculation of mean $\mathbf{k_{cat}/K_m}$ standard error. We did not use error propagation to calculate the standard errors of the 20 mean k_{cat}/K_m values provided in Table 3. The commonly used error propagation formula for calculating the standard error of a ratio of two quantities assumes the measurements used to compute the numerator are independent of those used to compute the denominator. Since each k_{cat} measurement is paired with a K_m measurement from the same Michealis-Menten fit, this assumption is suspect. The jackknife method does not assume that the k_{cat} and K_m measurements are independent. In addition, simulation studies indicate that for estimating the standard error of a ratio, the jackknife standard error estimate is superior to that provided by error propagation.[3] Therefore we computed the standard error of each mean k_{cat}/K_m ratio using the jackknife. Suppose our

data consist of n k_{cat}-K_m pairs: $\{(k_{cat,1}, K_{m,1}), \ldots, (k_{cat,n}, K_{m,n})\}$. Our estimate of the mean k_{cat}/K_m ratio is $r = \bar{k}_{cat}/\bar{K}_m$ where $\bar{k}_{cat}$ and $\bar{K}_m$ are the averages of the n k_{cat} and K_m measurements, respectively. The jackknife standard error estimate for r is then

$$\begin{aligned} \mathrm{SE}(r) &= \left[\frac{n-1}{n}\sum_{i=1}^{n}(r_i - \bar{r})^2\right]^{0.5} \\ &= \left[\frac{(n-1)^2}{n}S_r^2\right]^{0.5} \end{aligned}$$

where r_i is the value we get for r if we omit the ith k_{cat}-K_m pair, $\bar{r}$ is the mean of $r_1, \ldots, r_n$, and S_r^2 is the sample variance of $r_1, \ldots, r_n$.[4] For example, suppose our data consist of the three k_{cat}-K_m pairs: $(1, 2), (2, 3), (3, 4)$. Then $r_1 = (2+3)/(3+4) = 5/7$, $r_2 = 2/3$, $r_3 = 3/5$; and $S_r^2 = 0.0033$. Thus $\mathrm{SE}(r) = \sqrt{(4/3)(0.0033)} = 0.066$.

Hypothesis testing via z test. When comparing two means using estimated standard errrors, one typically uses a t test rather than a z test. First, we note that both tests use the same test statistic. For example for comparing $K_{m,1}$ and $K_{m,2}$ our test statistic is

$$z = t = \frac{\bar{K}_{m,1} - \bar{K}_{m,2}}{\sqrt{SE^2_{\bar{K}_{m,1}} + SE^2_{\bar{K}_{m,2}}}} = \frac{2.89 - 7.66}{\sqrt{0.318^2 + 0.833^2}} = -5.36$$

Since $\bar{K}_{m,1}$ and $\bar{K}_{m,2}$ are computed from large numbers of measurements (70 and 84, respectively), the degrees of freedom for the t distribution of the above test statistic will be large enough that, for our purposes, it will be indistinguishable from the standard normal (z) distribution. Thus, since estimating the degrees of freedom using a Satterthwaite-type approximation would be a tedious and difficult task, we used the standard normal distribution to compute the p-value, thus performing a z test instead of a t test. The p-value corresponding to the above z statistic of -5.36 is 8.3E-8.

(1) Navidi, W. (2011) *Statistics for Engineers and Scientists, 3rd Edition*, McGraw-Hill, page 170.

(2) Mandel, J. (1964) *The Statistical Analysis of Experimental Data*, Dover, page 60.

(3) Efron, B.(1982) *The Jackknife, the Bootstrap, and Other Resampling Plans*, SIAM, pages 16-17.

(4) Efron, B.(1982) *The Jackknife, the Bootstrap, and Other Resampling Plans*, SIAM, page 13.
